# Prevalence of coronavirus disease 2019 in a multiethnic cohort of patients with autoimmune rheumatic diseases in Qatar

**DOI:** 10.5339/qmj.2022.37

**Published:** 2022-08-05

**Authors:** Karima Becetti, Eman Satti, Betsy Varughese, Yousef Al Rimawi, Rawan Sheikh Saleh, Nawal Hadwan, Miral H Gharib, Mohamed Awni Al Kahlout, Essa Abuhelaiqa, Hadil Afif Ashour, Rajvir Singh, Samar Al Emadi

**Affiliations:** ^1^Division of Rheumatology, Department of Medicine, Hamad Medical Corporation, Doha, Qatar E-mail: becettikarima@gmail.com; ^2^Gastroenterology & Hepatology, Medicine, Hamad Medical Corporation, Doha, Qatar; ^3^Division of Nephrology, Department of Medicine, Hamad Medical Corporation, Doha, Qatar; ^4^Cardiology Research Center, Heart Hospital, Hamad Medical Corporation, Doha, Qatar

**Keywords:** Autoimmune disease, rheumatic disease, disease-modifying anti-rheumatic drugs, COVID-19, SARS-COV-2

## Abstract

Background: Autoimmune rheumatic diseases (ARDs) are characterized by immune dysfunction and associated with an increased risk of infections, which were of significant concern during the coronavirus disease 2019 (COVID-19) pandemic. Variable rates of COVID-19 incidence have been reported in patients with ARDs; however, the true effect of this infection on this patient population is still unclear. We, therefore, aimed to evaluate the COVID-19 prevalence among a multiethnic cohort of patients with ARDs in Qatar.

Material and Methods: We used telephonic surveys to collect demographic and clinical information of patients with ARD in Qatar between April 1 and July 31, 2020, including any close contact with a COVID-19 case at home or work and polymerase chain reaction (PCR)-confirmed COVID-19 diagnosis. An electronic medical records review was conducted to verify pertinent data collected through the surveys. Prevalence with 95% confidence interval (CI), Student's t-tests, and chi-square/Fisher's exact tests were used for univariate analyses, whereas multivariate logistic regression was used to identify factors associated with COVID-19.

Results: The study included 700 patients with ARD (mean age, 43.2 ± 12.3 years), and 73% were female. Until July 2020, 75 (11%, 95% CI 9%–13%) patients had COVID-19. Factors associated with COVID-19 included being a man (adjusted odds ratio [aOR] 2.56, 95% CI 1.35–4.88, *p* = 0.01) and having close contact with a COVID-19 case (aOR 27.89, 95% CI 14.85–52.38, *p* = 0.01). Disease severity and rheumatic medications had no significant association with the odds of contracting COVID-19. In the 86 patients with ARD having close contact, the frequency of hydroxychloroquine utilization was lower in patients who contracted COVID-19 than in those who did not (35% vs 72.5%, *p* = 0.01).

Conclusions: In Qatar, patients with ARDs had an overall higher prevalence of COVID-19 than global estimates. Being male and having close contact with a COVID-19 case were strongly associated with COVID-19 as reported globally. The presence of comorbid conditions, disease-specific factors, and rheumatic medications had no significant effect on the risk of COVID-19 in our study suggesting alternative mechanisms to the increased prevalence.

## Introduction

Since its discovery in Wuhan, China, in December 2019, severe acute respiratory syndrome coronavirus 2 (SARS-COV-2) has spread worldwide with a high transmission rate. On March 11, 2020, it was declared a pandemic by the World Health Organization (WHO)^1^. As of April 14, 2022, the WHO reported more than 500 million cases of coronavirus disease 2019 (COVID-19) in more than 197 countries and territories, resulting in more than 6 million deaths^2^.

Large descriptive studies from China, Europe, and the USA have identified risk factors for severe COVID-19, including being of older age and having some medical conditions such as diabetes, hypertension, and chronic kidney disease^3,4^. However, the effect of COVID-19 on patients with autoimmune rheumatic diseases (ARDs) is still unclear.

ARDs are rare diseases characterized by immune dysfunction and chronic inflammation. The prevalence of rheumatoid arthritis (RA), one of the more common ARDs, is estimated at 0.5%–1.0% worldwide^5^. In the Middle East and North Africa, a global burden study estimated a rheumatoid arthritis prevalence of 0.16% in 2010^6^. Infection is one of the leading causes of mortality in patients with ARDs. Patients with RA are at a higher risk of death from infections^7,8^ and of contracting nonfatal infections^9^ than the general population. Tektonidou et al. found that patients with systemic lupus erythematosus (SLE) had a hospitalization rate for serious infections that was more than 12 times higher than that of patients without SLE^10^. Similarly, Bosch et al. concluded that patients with SLE had an overall risk for infections (including pneumonia) that was approximately 60% higher than patients without SLE with no risk factors of infection^11^.

Multiple factors contribute to this increased infection risk, including the immune system dysfunction that is intrinsic to ARDs. Additionally, ARDs are frequently associated with chronic comorbidities such as diabetes and cardiovascular disease^12^, which increase the COVID-19 risk. ARDs are also commonly treated with immunosuppressant therapies, including glucocorticoids (GCs) which, among other adverse effects, are known to increase the risk of infection^13^. Contrastingly, several disease-modifying anti-rheumatic drugs (DMARDs), including hydroxychloroquine (HCQ), tocilizumab, baricitinib, and GCs, are being investigated and used in the management of COVID-19 because of their antiviral and immunomodulatory effects, as the most common cause of severe COVID-19 is cytokine storm syndrome^14^. Therefore, these medications might alter the susceptibility of patients with ARDs to COVID-19.

Thus far, variable COVID-19 infection rates in patients with ARDs have been reported in countries with different patterns of COVID-19 spread, ranging from 0.1% to 8%^15^. These rates also differed relative to that in the reference (general) population. Additionally, no previous studies have examined the rate of COVID-19 in patients with ARDs in countries of the Gulf Cooperation Council (GCC).

In this study, we aimed to estimate the prevalence and risk factors for COVID-19 in patients with ARDs during the pandemic's peak in Qatar, which has 2.8 million multinational populations and is one of the countries with the highest COVID-19 infection rates worldwide, estimated at more than 40,000 cases per 1 million populations in September 2020.

## Methods

### Study design and participants

The study was approved by the Medical Research Center and Institutional Review Board of Hamad Medical Corporation in Doha, Qatar (MRC-01-20-617).

We performed a cross-sectional survey of patients with ARDs who were residing in Qatar between April 1, 2020, and July 31, 2020. Purposive sampling was used to select participants from the repository of patients with ARDs regularly following up in the rheumatology clinics at hospitals across Hamad Medical Corporation. Eligible patients were invited to participate in a structured telephonic survey. Hamad Medical Corporation is the principal public health care provider in Qatar managing nine specialty and three community hospitals. During the pandemic, it was the sole provider of COVID-19 care across the country. To enhance recruitment, the study was also advertised through the hospital's social media ports to ensure widespread announcements in both high- and low-risk areas. Eligible patients had a physician-confirmed diagnosis of an ARD, including RA, SLE, Sjogren's syndrome (SS), antiphospholipid syndrome, mixed connective tissue disease, systemic sclerosis (SSc), inflammatory myositis, vasculitis, Behcet's disease, psoriatic arthritis, ankylosing spondylitis and other spondyloarthropathies, and undifferentiated connective tissue diseases. To participate in the study, patients had to be 18 years old or older, able to complete the telephone interview, and willing to provide verbal informed consent. The need for written informed consent was waived by the Institutional Review Board. A description of the research and its objectives, a statement clarifying the implications of the research on participants’ confidentiality, information on how the participant can obtain answers to pertinent research questions, and a statement about the voluntary nature of study participation were explained before the interview.

After obtaining verbal consent, one of the investigators completed the data collection sheet through a telephone interview. Pertinent clinical data, such as ARD diagnosis, medications, and COVID-19 PCR tests results were verified via electronic medical records review. After the interview, patients were provided with a hotline number to call if they had new contact with a patient having COVID-19 or if they were diagnosed with COVID-19. The collected data were updated accordingly. To unify the data collection process, investigators received training on the structured telephone survey, electronic chart review, and data entry before the start of the study.

#### Study variables:

Through telephone survey, we collected data on the patient demographics (including age, sex, ethnicity, and smoking status); chronic medical comorbid conditions including diabetes mellitus, hypertension, chronic kidney and heart disease, and malignancy among others; and non-ARD medications of interest, including angiotensin-converting enzyme inhibitors (ACE-Is) and angiotensin II receptor blockers (ARBs). ARD-specific data included the specific diagnosis; disease duration; disease activity based on the most recent rheumatology assessment in electronic medical records (remission vs. active); and most recent ARD medication regimens including GCs, nonsteroidal anti-inflammatory drugs (NSAIDs), and conventional, biologic, and targeted synthetic DMARDs. Patients were also asked whether they received the seasonal influenza vaccination. Data on any contact with patients having COVID-19 were obtained from the beginning of the pandemic and were considered positive if it occurred at home or in the workplace (close contact). In addition, patients were asked if they had ever been diagnosed with COVID-19 based on a PCR test. COVID-19 PCR testing was centrally performed at the study center in Qatar, which made it possible to confirm the testing and results after exposure via chart review.

### Statistical analysis

IBM SPSS Statistics for Windows, version 26.0 (IBM Corp., Armonk, NY, USA) was used for the analysis.

Descriptive analyses in the form of mean and standard deviation and frequency distribution with percentages for continuous and categorical variables, respectively, were used to describe patients’ characteristics and COVID-19 infection rate during the study period. Demographic, medical, and exposure variables were compared between the COVID-19 group and non-COVID-19 group using Chi-square or Fisher's exact tests and Student's t-test for categorical and continuous variables, respectively. Multivariate logistic regression analysis was subsequently used to identify variables independently associated with COVID-19. Stratified analysis was conducted in a subgroup of patients who had been exposed to a positive COVID-19 case. All tests were two-tailed, and a P-value < 0.05 was considered significant.

## Results

### Patient characteristics

We recruited 700 patients between April 12, 2020, and July 31, 2020 (mean age 43.2* ± *12.3 years old; 73% women; **Table 1**). RA and SLE were the most common autoimmune diseases among the recruited patients (37% and 22%, respectively). Approximately one-quarter of the patients (26%) had active disease at the time of the interview. HCQ was the most commonly used treatment, followed by methotrexate and TNF inhibitors (45%, 25%, and 18%, respectively). Only 16% of the patients were on GCs, which was defined as a regular treatment for more than 3 months regardless of the dose.

During the study period, which represented the first peak of the COVID-19 pandemic in Qatar, we identified 75 (11%, 95% CI 9%–13%) patients who tested positive for COVID-19 according to reverse-transcription PCR testing.

### Factors associated with COVID-19 in patients with ARDs

Sociodemographic and clinical variables were compared between patients with ARDs who contracted COVID-19 and those who did not (Table 1). Only ARDs with high prevalence (RA and SLE) were included in the univariate and multivariate analyses. Most of the patients with COVID-19 were men (33 [44%] vs. 155 [25%], *p* = 0.01), whereas age and ethnicity were not significantly different between the two groups. The prevalence of diabetes was higher in the COVID-19 group than in the non-COVID-19 group (18 [24%] vs. 87 [14%], *p* = 0.02), whereas obesity and SLE were more common in the non-COVID-19 group (*p* = 0.04 and *p* = 0.02, respectively). Furthermore, the number of patients with interstitial lung disease (ILD) was higher in the COVID-19 group than in the non-COVID-19 group (5 [7%] vs. 3 [0.5%], *p* = 0.01); however, the ILD frequency was too low to make an accurate conclusion regarding its association with COVID-19. Long-term treatment with HCQ was more prevalent in the non-COVID-19 group; however, this difference was not significant (26 [35%] vs. 288 [46%], *p* = 0.06). Similarly, mycophenolate mofetil use was significantly more prevalent in the non-COVID-19 group (1 [1%] vs 66 [11%], *p* = 0.01). No differences in other ARD medications, steroids, NSAIDs, and ACE-I/ARB were observed. Finally, a history of close contact with a patient having COVID-19 was significantly more common in the COVID-19 group than in the non-COVID-19 group (45 [60%] vs. 41 [7%], *p* = 0.01).

According to a subgroup analysis of patients who had close contact with COVID-19 cases (n = 86), 46 (53.5%) were on HCQ treatment. The number of patients with COVID-19 was lower among patients who were on HCQ treatment than in those who were not (16 [35%] vs 29 [72.5%], *p* = 0.01) (Flow Chart 1).

To identify factors independently associated with COVID-19, significant and clinically relevant variables in the univariate analysis were taken into consideration for the multivariate logistic regression analysis using binary outcomes of the COVID-19 group vs. non-COVID-19 group. Obesity and mycophenolate mofetil, along with ILD, were not included in the multivariate regression model because of the very low number of observations in the COVID-19 group and the uninterpretable 95% CI. The final model is presented in **Table 2**.

The COVID-19 risk was 2.5-fold higher in men than in women (adjusted odds ratio [aOR] 2.56, 95% confidence interval [CI] 1.35–4.88, *p* = 0.01). Regarding the medical and ARD-specific variables, the presence of diabetes as comorbidity resulted in a two-fold increase in COVID-19 risk with a tendency toward significance (aOR 2.14, 95% CI 1.0–4.6, *p* = 0.051). Moreover, a history of close contact with a COVID-19 case carried a 28-fold increase in COVID-19 risk (aOR 27.89, 95% CI 14.85–52.38, *p* = 0.01).

## Discussion

In this large cross-sectional study, we determined a COVID-19 prevalence of approximately 11% (95% CI 9%–13%) in patients with ARDs in Qatar. This prevalence was higher than the estimated prevalence in Qatar's general population with 101,911 confirmed COVID-19 cases and prevalence rate of 3.6% (95% CI 3.58%–3.62%) reported by the end of the study recruitment period^2^. It was also overall higher than the infection rates among patients with ARDs in other countries.

An earlier report from China showed a COVID-19 infection rate of 0.43% in 6228 patients with ARDs, which was higher than that reported in the general population (0.12%)^16^. In families with a member positive for COVID-19 included in this study, patients with ARDs had a higher COVID-19 infection rate than those without ARDs (63% vs. 34%, *p* = 0.0018). Subsequent reports showed significant variability in the COVID-19 infection rates in patients with ARDs and how they compared with the general population. In Spain, the incidence rate of hospital PCR-confirmed COVID-19 cases among patients with rheumatic diseases was estimated at 0.76%, compared with 0.58% in a reference population (odds ratio [OR] 1.32, 95% CI 1.15–1.52, *p* < 0.0001)^17^. A lower rate was reported in a SARS-CoV-2 registry in Hamburg, Germany, where 30 of 11,771 patients (or 0.25%) who were prescribed any DMARD contracted COVID-19 compared with an incidence of 0.28% in the general population, indicating that patients with rheumatic diseases on DMARDs were not at higher risk for COVID-19^18^. Survey studies from Italy reported COVID-19 infection rates ranging from 0.2% to 7.2% depending on the ARD population studied and the definition of COVID-19 used (confirmed vs. suspected)^19–24^. In a more recent study of a USA cohort of 4,666 patients with ARDs, 5.6% developed COVID-19 over a follow-up period of 9 months^24^. A meta-analysis of 62 observational studies found a pooled COVID-19 prevalence of 0.011 in patients with ARDs. This study also found that the COVID-19 risk was significantly higher in this patient population than in controls (OR 2.19, 95% CI 1.05–4.58, *?p* = 0.038)^15^.

The COVID-19 infection rate may also vary between different ARDs. In our study, SLE was less frequent in patients with COVID-19; however, after adjusting for other variables, no significant association was found between SLE and COVID-19. This was similar to the findings of a study from Spain where patients with SLE appeared to have a lower COVID-19 incidence than other connective tissue diseases, whereas SS, SSc, and polymyalgia rheumatica/giant cell arteritis had the highest incidences^17^. Contrastingly, SLE had a slightly higher COVID-19 prevalence in two survey studies from Italy: one that included 417 (1.2% vs. 0.73%) patients and the other that included 165 (7.2% vs. 0.76%) patients^25,26^. Pooling these studies together, 41 of 4307 patients (or 0.9%) with SLE had a PCR-confirmed COVID-19 diagnosis which slightly exceeded the prevalence in the general population^27^. On the contrary, patients with inflammatory arthritis were found to have a lower overall prevalence of suspected and confirmed COVID-19 than patients with connective tissue diseases including SLE, SS, SSc, and inflammatory myositis^28^. However, a more recent matched cohort study showed that patients with RA had a higher risk of COVID-19 than non-RA controls with an adjusted hazard ratio [HR] of 1.25 (95% CI 1.13–1.39)^29^.

In addition to ARD, other factors are suggested to affect the COVID risk in this population including age and non-autoimmune chronic conditions such as diabetes, which was more frequent in the COVID-19 group in our analysis. A study estimated that the unadjusted OR of testing positive for COVID-19 while having an ARD was 1.51 (95% CI 1.38–1.64), whereas the adjusted OR was 1.00 (95% CI 0.92–1.10) after accounting for these comorbidities^30^. ARD medications also have complex associations with COVID-19 prevalence from increased risk to potentially protective effects. In our study, HCQ treatment was more common in the non-COVID-19 group, especially after close contact with a COVID-19 case. This was also shown by Zhong et al. who found that patients who were taking HCQ had a lower COVID-19 risk (OR 0.09, 95% CI 0.01–0.94, *?*p = 0·044)^16^. However, trials on HCQ showed a lack of efficacy in treating COVID-19 and in post-exposure prophylaxis^31^. Other observational studies also did not show a significant association between HCQ and COVID-19^27,31–33^. With regard to DMARDs, our study was in concordance with previous studies that showed a similar COVID-19 incidence^17,15,34^ or an even lower COVID-19 prevalence^28^ in patients taking these medications. Despite the known increased infection risk with GCs^13^, their effect on the risk of COVID-19 development is not as well understood. Studies on populations with a higher proportion of GC use had a higher COVID-19 prevalence and GC exposure was associated with higher odds of COVID-19 infection^15,24^. Our data did not show an association between GC and COVID-19; however, this may be due to the low percentage of patients on GCs in our cohort (16%).

An interesting finding in our study was the predominance of men among patients with ARDs who contracted COVID-19. Globally, the COVID-19 infection rate appeared to be similar between men and women according to the COVID-19 Sex-Disaggregated Data Tracker^35^. However, our findings correlated with those from the study by Omrani et al., whose evaluation of the first 5,000 COVID-19 cases in Qatar showed higher infection rates among men and young adults^36^. Moreover, they found that men were twice as likely to contract COVID-19 in Qatar. This is thought to be due to the population composition where the male-to-female ratio was approximately 2.8^36^ and the number of male migrants working on the state's infrastructure development. This trend was also observed in other disease populations in Qatar such as solid-organ transplant recipients^37^.

Lastly, we showed that close contact with a COVID-19 case occurred in 60% of our COVID-19 cases. This finding carried the most significant association with COVID-19 in our cohort. This was also demonstrated in other studies of patients with ARDs^25^ and emphasizes the importance of preventive measures, including physical distancing, in decreasing the COVID-19 risk, as strongly recommended by rheumatology societies worldwide^38,39^.

To the best of our knowledge, this study is the first to report on the COVID-19 infection rate in patients with ARDs in the GCC. The large sample size and number of variables examined were important strengths. Our cohort included patients with multiple rheumatic diseases that allowed for the examination of the COVID-19 risk in more prevalent diseases such as SLE and RA. Since the study center is the largest medical provider in the country, this cohort is reflective of the general population of patients with ARDs in Qatar. Advertising on social media allowed for the identification of patients from other centers as well. Another important strength is that all COVID-19 cases were confirmed by PCR testing, which was completed centrally at the study center. This made it difficult to overlook patients in our cohort who were COVID-19 positive. Moreover, the country's policy on extensive contact tracing and massive testing allowed for the identification of asymptomatic cases and for patients to be confirmed as negative after contact with a positive case. It was also possible for the study participants to call back and report on any new sick contact or if they tested positive for COVID-19, which may have enriched our cohort with COVID-19 patients.

When interpreting the results, some limitations should be recognized. A survey study is dependent on participants’ recollection of information, such as contact with COVID-19 cases and influenza vaccinations. Variables such as disease activity, an important factor when assessing infection risk, were difficult to discern using these phone surveys. Additionally, as stated earlier, the population in Qatar is unique in its composition and has a higher prevalence of men than women, which may limit the generalizability of the results. In addition, due to a lack of consensus on ethnicity classification in Qatar, the patient's self-reported country of origin was used to determine ethnicity potentially limiting the accurate assessment of the effect of ethnicity on the risk of COVID-19 in patients with ARDs in our study. Most importantly, an accurate evaluation of whether having an ARD independently increased COVID-19 risk was not possible in our study due to the lack of a control group.

## Conclusion

In a multiethnic population in the GCC, we found that patients with ARDs had a COVID-19 prevalence of 11%. This prevalence is higher than the overall global estimates, does not appear to be driven by disease-specific variables, medications or comorbidities, and adds to the substantial variability in the reported COVID-19 prevalence in patients with ARDs worldwide. The lack of a better understanding of the true risk patients with ARDs face during the pandemic creates significant uncertainty for patients and managing rheumatologists and continues to pose a challenge even after the introduction of effective vaccines against COVID-19. Both large international studies and local countrywide data are needed to better inform management recommendations and guide changes in patients’ behavior and perceptions. Future studies should aim to evaluate the outcomes of COVID-19 in patients with ARDs in Qatar and determine risk factors for poor outcomes.

## Figures and Tables

**Flow Chart 1.**
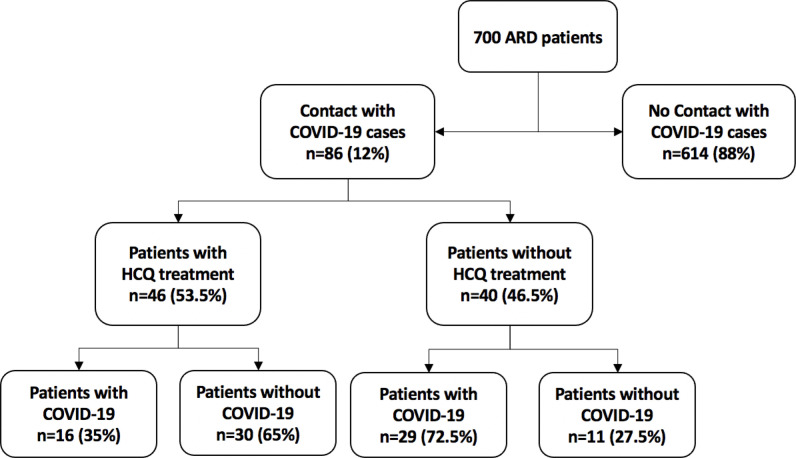
Subgroup analysis of the 86 patients who had close contact with a COVID-19 case ARD, autoimmune rheumatic disease; COVID-19, coronavirus disease 2019; HCQ, hydroxychloroquine

**Table 1:** Characteristics of patients with ARDs and differences in demographic and clinical variables between the COVID-19 and non-COVID-19 groups*

Variables	All patients with ARDs (n=700)	COVID-19 (n=75)	Non-COVID-19 (n=625)	*p*

Age (years)	43.2 ± 12.3	42.8 ± 12.6	43.2 ± 12.3	0.77

Sex (male)	188 (27%)	33 (44%)	155 (25%)	0.01

*Ethnicity*				0.07

Asian	266 (38%)	39 (52%)	227 (36%)	

Levant	73 (10%)	7 (9%)	66 (11%)	

Gulf	191 (27%)	16 (21%)	175 (28%)	

African	147 (21%)	13 (17%)	134 (21%)	

Others	23 (3%)	0 (0%)	23 (4%)	

*Comorbid conditions*				

Hypertension	148 (21%)	19 (25%)	129 (21%)	0.35

Diabetes	105 (15%)	18 (24%)	87 (14%)	0.02

Obesity	79 (11%)	3 (4%)	76 (12%)	0.04

Chronic kidney disease	33 (5%)	3 (4%)	30 (5%)	0.76

Chronic heart disease	18 (3%)	3 (4%)	15 (2%)	0.41

Interstitial lung disease	8 (1%)	5 (7%)	3 (0.5%)	0.01

*Type of ARD*				

RA	260 (37%)	34 (45%)	226 (36%)	0.12

SLE	151 (22%)	8 (11%)	143 (23%)	0.02

Sjogren’s syndrome	59 (8%)	-	-	

APLS	39 (6%)	-	-	

Other CTDs^*^^*^	25 (4%)	-	-	

Ankylosing spondylitis	75 (11%)	-	-	

Psoriatic arthritis	41 (6%)	-	-	

Other SpA	15 (2%)	-	-	

Behcet’s disease	10 (1%)	-	-	

*ARD medications*				

Glucocorticoids	114 (16%)	14 (19%)	100 (16%)	0.56

NSAIDs	32 (5%)	5 (7%)	27 (4%)	0.36

Hydroxychloroquine	314 (45%)	26 (35%)	288 (46%)	0.06

Mycophenolate mofetil	67 (10%)	1 (1%)	66 (11%)	0.01

Methotrexate	173 (25%)	25 (33%)	148 (24%)	0.07

Azathioprine	48 (7%)	8 (11%)	40 (6%)	0.17

Sulfasalazine	60 (9%)	5 (7%)	55 (9%)	0.53

Leflunomide	26 (4%)	3 (4%)	23 (4%)	0.89

Anti-TNF	124 (18%)	13 (17%)	111 (18%)	0.93

JAKi	26 (4%)	1 (1%)	25 (4%)	0.25

Rituximab	33 (5%)	5 (7%)	28 (5%)	0.4

Cyclophosphamide	1 (0.1%)	0 (0%)	1 (0.2%)	0.73

Tocilizumab	11 (2%)	0 (0%)	11 (2%)	0.25

ACEi/ARB	37 (5%)	5 (7%)	32 (5%)	0.57

*Disease activity*				0.24

Remission	516 (74%)	51 (68%)	465 (74%)	

Active	184 (26%)	24 (32%)	160 (26%)	

Flu vaccination	299 (43%)	30 (40%)	269 (43%)	0.62

Close contact with a COVID-19 case	86 (12%)	45 (60%)	41 (7%)	0.01


* Values are presented as number (%) and mean (SD) for categorical and continuous variables, respectively.

** Other CTDs included systemic sclerosis, mixed connective tissue disease, undifferentiated connective tissue disease, and idiopathic inflammatory myositis.

ACEi/ARB, angiotensin-converting enzyme inhibitors/angiotensin II receptor blockers; anti-TNF, anti-tumor necrosis factor; APLS, antiphospholipid syndrome; ARD, autoimmune rheumatic disease; COVID-19, coronavirus disease 2019; CTD, connective tissue disease; JAKi, Janus kinase inhibitor; NSAIDs, nonsteroidal anti-inflammatory drugs; RA, rheumatoid arthritis; SLE, systemic lupus erythematosus; SpA, spondyloarthritis.

**Table 2:** Factors independently associated with COVID-19 in patients with ARDs

Variable	Adjusted odds ratio (aOR)	95% confidence interval (CI)	*p*

**Age (years)**	0.99	0.96–1.01	0.28

**Sex (male)**	2.56	1.35–4.88	0.01

**Diabetes**	2.14	1.00–4.60	0.051

**SLE**	0.57	0.22–1.50	0.25

**Hydroxychloroquine**	0.60	0.30–1.24	0.17

**Close contact with a COVID-19 case**	27.89	14.85–52.38	0.01


COVID-19, coronavirus disease 2019; SLE, systemic lupus erythematosus; ARDs, autoimmune rheumatic diseases.
